# Supplementary material for: Overexpression of VEGF in the MOPC 315 Plasmacytoma Induces Tumor Immunity in Mice
Source: Int J Mol Sci. 2022 May 7;23(9):5235. doi: 10.3390/ijms23095235 (PMC9104487; doi:10.3390/ijms23095235)
Supplement: Supplementary file 1 [file ijms-23-05235-s001.zip › ijms-1697340-supplementary.pdf]

**Supplementary Figure S1**

A

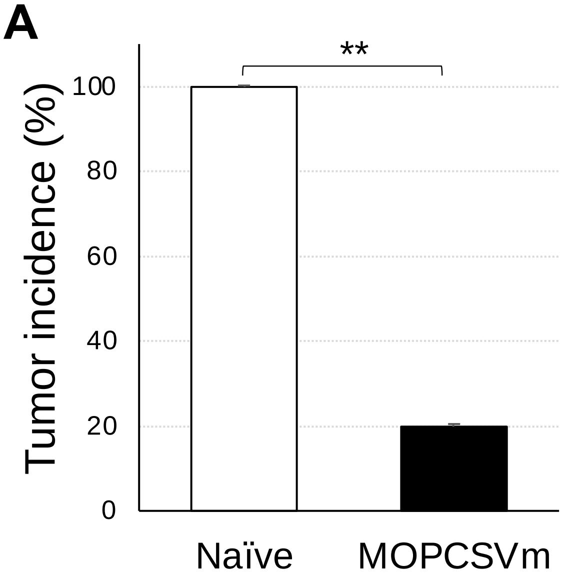


**Supplementary Figure S1.** (**A**) Memory of the CTL response in mice pre-challenged with MOPCSVm was demonstrated by inoculating the parental MOPC 315 cells into naïve mice or into recipients of MOPCSVm cells in which tumors had regressed. ** $p < 0.01$.

**Supplementary Figure S2**

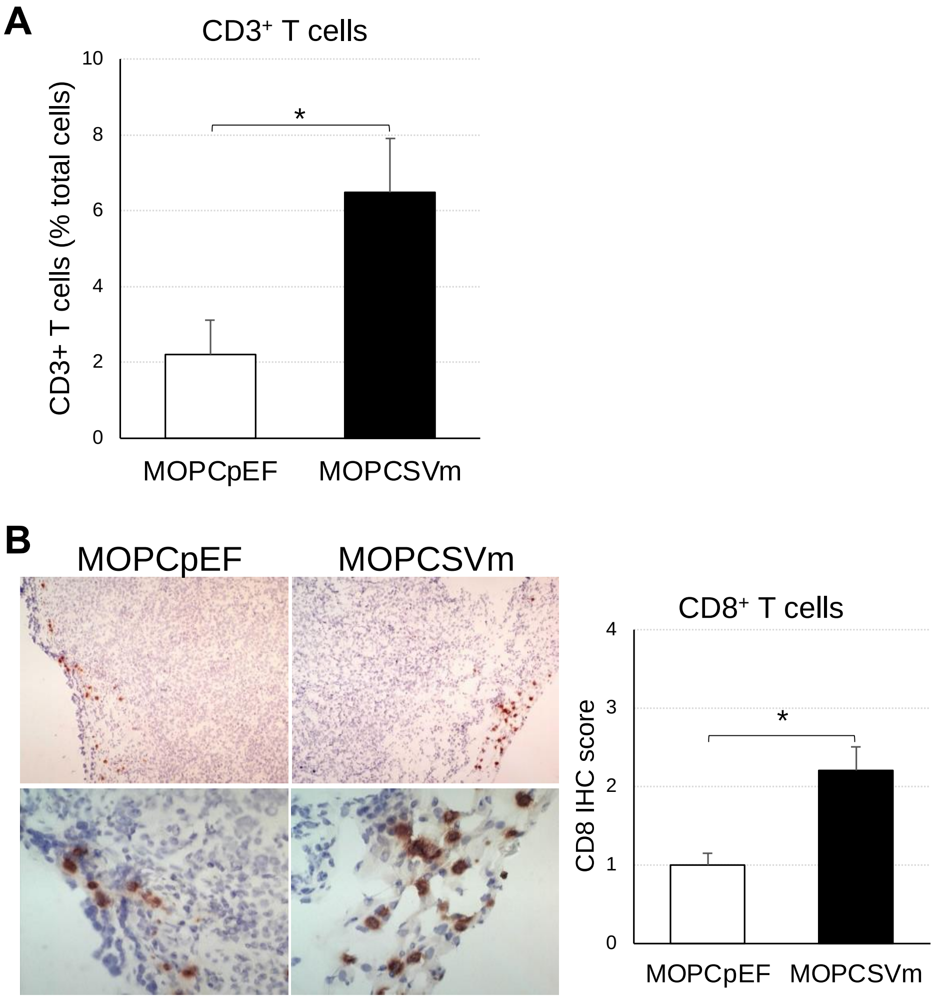


**Supplementary Figure S2.** T cell infiltration in tumor sites. (**A**) Flow cytometry analysis for tumor infiltrating lymphocytes. Flow cytometry analysis was performed to confirm whether $CD3^+$ T cell number were affected by VEGF produced from MOPC 315 tumor cells. (**B**) IHC staining analysis for $CD8^+$ T cell infiltration in both MOPCpEF and MOPCSVm tumor sites. $p < 0.05$.

**Supplementary Table S1**

**Supplementary Table S1.** Modulation of antitumor immune response by varying the concentration of VEGF at MOPC 315 plasmacytoma and B16F10 melanoma tumor site.

Modulation of antitumor immune response by varying the concentration of VEGF at tumor site

| Tumor cell | Treatment | Production of VEGF | *In vivo* tumor immune response | |
|---|---|---|---|---|
| | | | CTL | Tumor regression |
| MOPC 315 plasmacytoma | MOPCpEF (control) | 850pg/$10^5$ cells | 32% | 0% |
| | MOPCASVm (antisense) | 710pg/$10^5$ cells | 35% | 20% |
| | MOPCSVm (sense) | 20000pg/$10^5$ cells | 48% | 50% |
| | | | CTL | Tumor size |
| B16F10 melanoma | B16F10pEF (control) | 250pg/$10^5$ cells | 10% | 100% |
| | B16F10ASVm (antisense) | 190pg/$10^5$ cells | 10% | 85% |
| | B16F10SVm (sense) | 3000pg/$10^5$ cells | 10% | 140% |
